# Digital storytelling in health professions education: a systematic review

**DOI:** 10.1186/s12909-018-1320-1

**Published:** 2018-09-10

**Authors:** Katherine A. Moreau, Kaylee Eady, Lindsey Sikora, Tanya Horsley

**Affiliations:** 1 Faculty of Education, University of Ottawa, 145 Jean-Jacques-Lussier Private, Ottawa, ON K1N 6N5 Canada; 2 Health Sciences Library, University of Ottawa, 451 Smyth Road, Ottawa, ON K1H 8M5 Canada; 3 Research Unit, Royal College of Physicians and Surgeons of Canada, 774 Echo Drive, Ottawa, ON K1S 5N8 Canada

**Keywords:** Systematic review, Digital storytelling, Health professions education

## Abstract

**Background:**

Digital stories are short videos that combine stand-alone and first-person narratives with multimedia. This systematic review examined the contexts and purposes for using digital storytelling in health professions education (HPE) as well as its impact on health professionals’ learning and behaviours.

**Methods:**

We focused on the results of HPE studies gleaned from a larger systematic review that explored digital storytelling in healthcare and HPE. In December 2016, we searched MEDLINE, EMBASE, PsycINFO, CINAHL, and ERIC. We included all English-language studies on digital storytelling that reported at least one outcome from Levels 2 (learning) or 3 (behaviour) of The New World Kirkpatrick Model. Two reviewers independently screened articles for inclusion and extracted data.

**Results:**

The comprehensive search (i.e., digital storytelling in healthcare and HPE) resulted in 1486 unique titles/abstracts. Of these, 153 were eligible for full review and 42 pertained to HPE. Sixteen HPE articles were suitable for data extraction; 14 focused on health professionals’ learning and two investigated health professionals’ learning as well as their behaviour changes. Half represented the undergraduate nursing context. The purposes for using digital storytelling were eclectic. The co-creation of patients’ digital stories with health professionals as well as the creation and use of health professionals’ own digital stories enhanced learning. Patients’ digital stories alone had minimal impact on health professionals’ learning.

**Conclusions:**

This review highlights the need for high-quality research on the impact of digital storytelling in HPE, especially on health professionals’ behaviours.

**PROSPERO registration number:**

CRD42016050271.

**Electronic supplementary material:**

The online version of this article (10.1186/s12909-018-1320-1) contains supplementary material, which is available to authorized users.

## Background

Advances in technology increase the potential use of digital storytelling in health professions education (HPE). Digital storytelling combines stand-alone and first-person narratives with multimedia (e.g., images, music, narration, animation) to create 3–5 min videos [1–3]. Digital storytelling shares individuals’ lived experiences in ways that traditional storytelling (i.e., oral, written stories) cannot. Individuals can archive, retrieve, and review digital stories offline, as well as distribute them online to infinite audiences through websites or social media. With the inclusion of multimedia, digital stories can also effectively set and preserve the scenes and moods of individuals’ narratives [1, 4].

Those training and working across health sectors and disciplines, including individuals with minimal technological expertise, can create digital stories [5–7]. The creation and use of these stories can promote creative and reflective learning across health professions [8]. It can expose health professionals to others’ experiences, cultures, and viewpoints [2, 9]. It can also bring patients’ experiences and authentic voices into HPE and thus, potentially improve clinician-patient interactions as well as promote humanism and empathy in healthcare [2, 10].

To date, researchers have written commentaries and theoretical papers explaining the intricacies of creating and using health professionals’ as well as patients’ digital stories in a range of health sectors [8, 11, 12]. There are also some reviews that allude to or focus on digital storytelling. For instance, there is: (a) a meta-narrative review that groups all story forms (e.g., oral, written, digital) together to explore how storytelling can promote a culture change in healthcare organizations [13], (b) a literature review that focuses on the impact of digital storytelling in Kindergarten to Grade 12 [14], (c) a literature review that elucidates the therapeutic effects of digital storytelling among pediatric patients with cancer [7], (d) a scoping review that maps digital storytelling use in mental health, with a primary focus on patient/consumer education [15], and (e) a systematic review of the benefits and limitations of using digital storytelling in research within any context [16]. While these reviews describe digital storytelling, including its history, as well as its strengths and weaknesses, they do not focus on the contexts and purposes for using digital storytelling in HPE or its impact on health professionals’ learning and behaviours; areas of investigation that are critically important to fully understand and advance the use of digital storytelling in HPE. Thus, in the present study, we conducted a systematic review of empirical research examining digital storytelling in HPE to examine the contexts and purposes for using digital storytelling in HPE as well as its impact on the learning and behaviours of health professionals. We sought to answer the following questions:

1. In what contexts and for what purposes is digital storytelling used in HPE?
2. What impact does digital storytelling have on the learning and behaviours of health professionals?

## Methods

This paper presents results of HPE studies gleaned from a larger systematic review that explored the use and impact of digital storytelling in both healthcare and HPE. Adhering to the Preferred Reporting Items for Systematic Reviews and Meta-Analyses (PRISMA) guidelines [17], we systematically reviewed records that included relevant outcomes on the use of digital storytelling in HPE.

### Search strategy and study selection

**Fig. 1:**
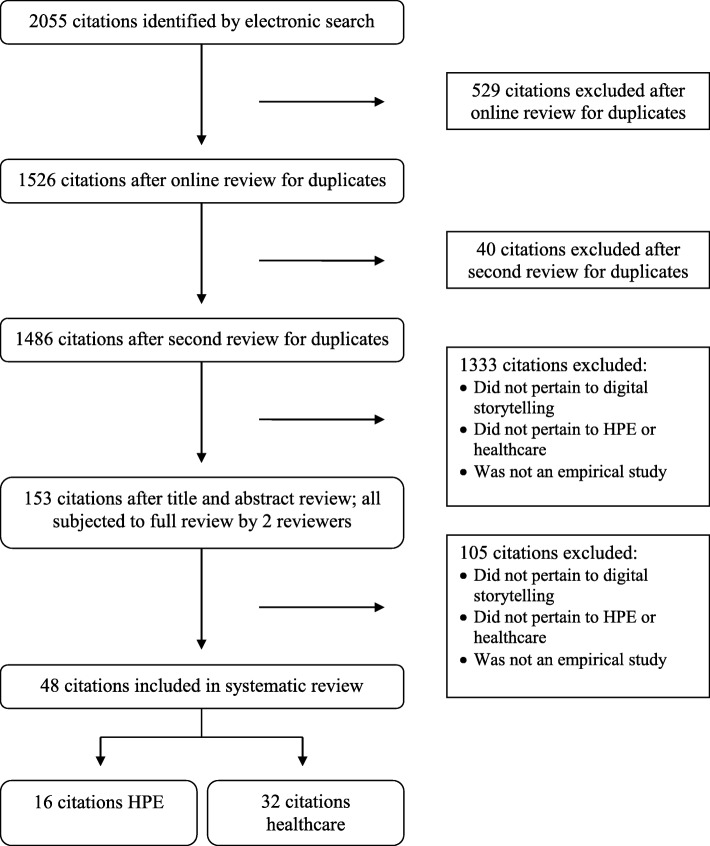
Systematic Review and Selection Process

Figure 1 presents our review and selection process. We collaborated with a health sciences librarian (LS) experienced in the development and conduct of systematic review searches to develop search strategies. In December 2016, we searched MEDLINE (see Additional file 1) from inception and tailored the search to EMBASE, PsycINFO, CINAHL, and ERIC. Two team members (KE & EM) independently reviewed the study titles and abstracts to remove duplicates and identify those that were potentially eligible. They then read the full texts to confirm eligibility and, when applicable, documented reasons for ineligibility. Given the complexity of the topic searched, the team hand-searched the reference lists of the included studies to identify those missed by the electronic search.

### Inclusion and exclusion criteria

We included all English-language studies on digital storytelling that met inclusion criteria and segmented those focused within the milieu of HPE for the purposes of this analysis. We included all levels of health professionals (e.g., student to practicing clinician), all types of health professionals (e.g., allied health professionals, nurses, physicians), and studies that focused on any type of educational setting. All included studies needed to report at least one outcome from Levels 2 or 3 of The New World Kirkpatrick Model [18]. These levels included: health professionals’ learning (i.e., degree of acquired knowledge, skill, attitude, confidence, and commitment) and health professionals’ behaviour (i.e., degree of change in behaviour). We limited the review to full-text articles of empirical studies. We did not place limitations on the date of the publication or the geographical location of the study. We excluded editorials, commentaries, literature reviews, as well as grey literature.

### Quality assessment

Two team members (KE & KM) independently assessed study quality of the retained studies. They used the Joanna Briggs Institute Critical Appraisal tools [19] to assess experimental, quasi-experimental, and qualitative studies. They used the Center for Evidence-Based Management Critical Appraisal tool [20] to assess non-experimental studies. In the case of mixed methods studies, they used the Center for Evidence-Based Management Critical Appraisal tool [20] to assess the quantitative portion and the appropriate Joanna Briggs Institute tool [19] to assess the qualitative portion. We did not use the quality assessment scores to exclude any studies. Instead, we used them as a point of discussion in the present article.

### Data extraction and analysis

We developed the data extraction sheet a priori and pilot tested it until we achieved sufficient duplicate agreement between two of the team members (KE & KM). Two reviewers (KE & KM) then independently extracted data from the included articles. They extracted the following information: publication characteristics, study objective(s), study design, setting, participant characteristics, digital storytelling characteristics, outcomes measured, and findings reported. They compared extractions and resolved differences through discussion or with a third non-author. They then analyzed the extracted quantitative data as well as the quality assessment data using descriptive statistics (i.e., frequencies and percentages) and the extracted qualitative data using qualitative content analysis.

## Results

### Characteristics and quality of the included studies

The comprehensive search (i.e., digital storytelling in both healthcare and HPE) resulted in 1486 unique titles and abstracts. Of these articles, 153 (10.3%) were eligible for full review, where 111 (72.5%) pertained to healthcare and 42 (27.5%) to HPE. From the 153 articles, 48 (31.4%) articles were suitable for data extraction. Of these studies, 32 (66.7%) focused solely on the use of digital storytelling in healthcare (reported elsewhere), with the remaining 16 (33.3%) focused on digital storytelling in HPE, which are the focus of this paper. As illustrated in Additional file 2, the HPE studies were published between 2004 and 2016, with an influx of studies in 2013 (*n* = 3, 18.8%), 2015 (*n* = 3, 18.8%), and 2016 (*n* = 4, 25.0%). Study design was variable including qualitative (*n* = 6, 37.5%), non-experimental (*n* = 4, 25.0%), mixed-methods (*n* = 4, 25.0%), quasi-experimental (*n* = 1, 6.3%), and a randomized-control trial (*n* = 1, 6.3%). As shown in Table 1, the quality of the studies varied, with most being of low (*n* = 8, 50.0%) to moderate quality (*n* = 5, 31.3%). Most of the studies were from the US (*n* = 8, 50.0%) or United Kingdom (*n* = 5, 31.3%), and only a few were from Canada (*n* = 2, 12.5%) and Australia (*n* = 1, 6.3%).

**Table 1:** Quality Assessment Scores of Included Articles

Author, year by study type	Critical appraisal tool	Score
Experimental		
Bruno [27]	JBI Checklist for Randomized-Controlled Trials	4/13
Quasi-Experimental		
Gazarian [25]	JBI Checklist for Quasi-Experimental Studies	6/9
Non-Experimental		
Cueva [32]	CEBM Critical Appraisal of a Survey	4/12
D’Alessandro [21]	CEBM Critical Appraisal of a Survey	2/12
Fenton [34]	CEBM Critical Appraisal of a Survey	3/12
Hewson [31]	CEBM Critical Appraisal of a Survey	2/12
Levett-Jones [26]	CEBM Critical Appraisal of a Survey	2/12
Qualitative		
Christiansen [28]	JBI Checklist for Qualitative Research	8/10
Loe [30]	JBI Checklist for Qualitative Research	5/10
Stacey [29]	JBI Checklist for Qualitative Research	3/10
Taylor [22]	JBI Checklist for Qualitative Research	9/10
Walsh [23]	JBI Checklist for Qualitative Research	9/10
Mixed-Methods		
Cueva [33]	CEBM Critical Appraisal of a Survey & JBI Checklist for Qualitative Research	3/12; 6/10
Eggenberger [24]	CEBM Critical Appraisal of a Survey & JBI Checklist for Qualitative Research	8/12; 5/10
Price [9]	CEBM Critical Appraisal of a Survey & JBI Checklist for Qualitative Research	7/12; 5/10
Snelgrove [35]	CEBM Critical Appraisal of a Survey & JBI Checklist for Qualitative Research	3/12; 9/10

### Contexts and purposes for using digital storytelling in HPE

Twelve (75.0%) studies focused on the use of digital storytelling at the undergraduate level, three (18.8%) concentrated on its use in continuing professional development (CPD), and one (6.3%) study described its use at the graduate level. Half of the studies involved undergraduate nursing students (*n* = 8, 50.0%). The remaining studies focused on social work (*n* = 2, 12.5%), medicine (*n* = 2, 12.5%), community health workers (*n* = 1, 6.3%), community health aids (*n* = 1, 6.3%), midwives (*n* = 1, 6.3%), and undergraduate students with general interests in health careers (*n* = 1, 6.3%).

The purposes for using digital storytelling in HPE were eclectic and included the teaching of: (a) clinical skills [21, 22]; (b) diversity, oppression, and social justice issues [23]; (c) concepts related to general patient support or person- patient-, and family-centered care [24–26]; (d) care provision for marginalized and underserved populations [27]; (e) transitions from student to working professional as well as development of professional identity [28, 29]; (f) intergenerational learning or social topics related to aging [30, 31]; (g) palliative care concepts [9]; and (h) care provision for those with chronic health conditions [32–35].

### Impact of digital storytelling on health professionals’ learning and behaviour

All 16 studies explored and reported the perceived impact of digital storytelling on the learning of health professionals using one or two of the following data collection strategies: self-reported questionnaires, standardized questionnaires, focus groups, or interviews. Of these 16 studies, five (31.3%) focused exclusively on health professionals’ learning from the creation of their own digital stories as part of a formal educational experience [9, 23, 25, 32, 33]. In these five studies, digital storytelling positively impacted the health professionals’ learning. For example, in Price et al. [9], nursing students created digital stories about palliative and end-of-life care from their professional experiences or from personal stories of their family members who experienced palliative and end-of-life care. They found that this creation experience increased their understanding of course concepts in ways that hypothetical case studies did not: (a) it taught them about the importance of making interpersonal connections through listening to others’ stories; (b) it facilitated their learning on cultural and spiritual awareness; and lastly, (c) it solidified the notion that each palliative or end-of-life care experience is unique [9]. Likewise, in the studies by Cueva and colleagues (2016, 2013) community health aids/practitioners and community health workers created digital stories about cancer. In the creation of these stories, health professionals drew on their own personal experiences with cancer, as well as factual knowledge about the disease. They found that the creation experience, along with their abilities to share their digital stories with their family members and communities, motivated them to increase their own knowledge and understanding of cancer, as well as changed their attitudes towards cancer and their reactions to those who had experienced it [32, 33]. Gazarian, Fernberg, and Sheehan [25], on the other hand, asked nursing students to create a digital story about an ethical concern encountered in practice. This approach was similar to that presented in Walsh, Shier, Sitter, and Sieppert [23], as they required graduate social work students to create digital stories on a diversity and oppression issue. In both studies, it was unclear if the students created digital stories based on personal experiences with the topic, if the concern/issue was something they witnessed in practice, or if it was a concept(s) they had explored in course material. Regardless, the students reported that the creation of the digital stories enriched and diversified their learning on the concerns/issues and allowed them to self-reflect.

Out of the 16 studies, three (18.8%) described health professionals’ learning from the perspectives of both the creators and viewers of the digital stories [29–31]. In these studies, the impact of digital stories on health professionals’ learning was, again, positive. For example, Stacey and Hardy [29] reported on the creation and use of newly qualified/practicing nurses’ digital stories to assist nursing students with their transitions to practice. The authors detailed how the qualified/practicing nurses learned about the intricacies of creating digital stories and how the creation process enhanced their reflective practices and emotional awareness in regards to experiencing professional transitions. In addition, they reported that the nursing students who viewed the digital stories learned that other nurses experienced similar concerns to their own about transitioning to practice. The authors also described how the newly qualified/practicing nurses’ stories encouraged the nursing students to reflect on how they might respond to transition challenges recounted in the digital stories [29]. Hewson, Danbrook, and Sieppert [31] and Loe [30] each explored the educational value of: (a) having students co-create digital stories with older adults, and (b) having students view older adults’ digital stories as a means for educating them about intergenerational learning and aging, respectively. In these studies, the co-creation of digital stories, as well as their viewing for educational purposes, enhanced the students’ knowledge about working with and caring for older adults, as well as the concept of ageism.

Five (31.3%) of the 16 studies concentrated on health professionals’ learning from listening to authentic patients’ digital stories [22, 27, 28, 34, 35]. Fenton [34] reported how a patient’s digital story increased nursing students’ understanding of a young person’s perspective of living with cancer and provided them with information about effective communication skills. Conversely, the other four studies reported that patients’ digital stories had minimal impact on health professionals’ understandings or knowledge of the given topics [22, 27, 28, 35]. That said, the authors highlighted that patients’ digital stories evoked emotional reactions among students that motivated them to potentially take action on the topics in future. For example, Bruno et al. [27] found that patients’ digital stories did not heavily influence medical students’ attitudes towards patients who are underserved, as the majority of the students were already sympathetic to this population. Nonetheless, the digital stories used in the study did increase students’ reported desires to provide care to underserved patients in future [27]. Taylor and Hutchings [22] also found that midwifery students did not acquire new breastfeeding knowledge from women’s digital stories on their breastfeeding experiences, but that the stories taught students how to better support breastfeeding women and evoked emotions among them to improve and advocate midwifery practice in regards to breastfeeding. Additionally, Christiansen [28] found that the extent to which nursing students learned from patients’ digital stories varied. In particular, while some students reported emotional reactions to, or emotional connections with the patients’ digital stories, that enhanced their learning on how to care for patients, other students thought that the digital stories were not as powerful as learning directly from patients in the classroom, or that the multimedia aspects (e.g., sound, pictures) overshadowed the patients’ key messages [28]. Moreover, Snelgrove, Tait, and Tait [35] found that although the nursing students valued the authenticity of the patients’ digital stories, some students did not recognize the intended link between the digital stories and the psychological course concepts being taught. Snelgrove et al. [35] also found that only a quarter of the students believed that the digital stories made them feel more confident in their abilities to care for patients with chronic illness.

In two (12.5%) of the 16 studies, educators used patients’ stories as inspiration for the creation of digital stories [21, 26]. More specifically, Levett-Jones, Bowen, and Morris [26] described the use of a virtual make-believe community that included the digital stories of ‘patients’. D’Alessandro, Lewis, and D’Alessandro [21] reported on an educational initiative that involved interviewing and photographing patients/family members and completing chart reviews on the patients. A second year medical student then used the collected information to create digital stories about the patients, which a practicing pediatrician and radiologist reviewed for accuracy. In both these cases, students reported increases in their learning about various health conditions and situations, which they thought would enhance their provision of care [21, 26].

Only one (6.3%) of the 16 studies explored, among other things, practicing nurses’ learning of family-centered care as a result of viewing digital stories from both health professionals and patients/family members [24]. In this article, the nurses perceived that their knowledge of family-centered care increased as a result of viewing the digital stories. They also thought that the stories increased their confidence in providing family-centered care [24].

Of note, three (18.8%) of the included studies also described how training individuals in the creation of digital stories positively impacted the creators’ or viewers’ learning from the digital stories themselves [23, 31, 36]. These training workshops ranged 3–5 days long and covered the processes of creating effective digital stories. Moreover, in another three (18.8%) studies, the authors commented on the educational benefits of including reflection activities, peer support, or additional guided learning following the use of patients’ digital stories [22, 28, 35].

Lastly, out of the 16 included studies, two (12.5%) focused on the perceived impact of digital storytelling on health professionals’ behaviours [32, 33]. The authors of these studies surveyed participants after completing a cancer education digital storytelling course. The participants in both studies reported that the creation of their digital stories lead them to take better care of themselves, their patients, their families, or their communities in order to decrease cancer risks (e.g., quit smoking, have a screening exam, eat healthier, increase physical activity) [32, 33].

## Discussion

This systematic review focused on the contexts and purposes for which digital storytelling was used in HPE as well as its impact on health professionals’ learning and behaviour. It showed that research on digital storytelling in HPE is still in an emerging phase with limited amounts of empirical literature on the topic. Given the popularity of social media platforms, such as Instagram (which we argue presents opportunities for informal/short digital stories), we speculate that HPE will continue to embrace and study digital storytelling. At present, this review reveals that the majority of digital storytelling studies are occurring in the undergraduate nursing context. This finding may reflect the emphasis that nursing often places on lived experiences, the use of constructive frameworks, and collaborative learning in educational practices [37]. However, there is also growing interest in arts-based pedagogy, which encompasses digital storytelling, in medicine as well as other health professions [38]. This pedagogy “use[s] art as a medium to support knowledge development in subjects other than art” [39]. Students can create art (e.g., digital stories) or respond to that of others to learn subject-specific topics. Research shows that such pedagogy can foster self-awareness, cultural awareness, community partnerships, social integration, observational skills, and whole person development [40]. Thus, as interest in arts-based pedagogy increases, we are hopeful that digital storytelling will become more prevalent in all health professions.

An important question raised by our findings is: what constitutes or counts as digital storytelling? As mentioned in the introduction, the definition of digital storytelling requires that it combines stand-alone and first-person narratives with multimedia. Individuals, especially those who believe in the importance of people retaining control over their own stories [2], may interpret this definition to imply that health professionals or patients need to create and tell their own digital stories, in order for them to be digital stories. However, as seen in Levett-Jones, Bowen, and Morris [26] and D’Alessandro, Lewis, and D’Alessandro [21], it is possible for someone else to create a stand-alone and first-person narrative that represents other individuals’ stories. Some may question whether this practice is appropriate. Thus, we suggest not only further research on the definition of digital storytelling, but also on the positive, negative, and ethical consequences of creating and using digital stories in HPE that portray others’ stories. Nonetheless, this review found that the creation of ‘patients’ stories’ by a third party appears to enhance health professionals’ learning.

The early data for the co-creation of patients’ stories alongside health professionals, as well as the creation and use of health professionals’ own digital stories suggest that it is more beneficial in terms of learning rather than the sole viewing of patients’ digital stories by health professionals. This review showed that in 4 (80.0%) out of 5 studies that concentrated on the use of patients’ digital stories in HPE, patients’ stories had minimal impact on health professionals’ understandings or knowledge of the given topics [22, 27, 28, 35]. Such findings provide some justification as to why other researchers have found that it is more common for health professionals to create their own digital stories than to listen to those of patients [2]. However, these four studies did discuss that patients’ digital stories induced emotional reactions among health professionals that motivated them to take on advocacy-type roles in future [22, 27, 28, 35]. These findings are minimally consistent with the literature on active patient involvement in HPE, which suggests that in addition to experiencing increased sensitivity and empathy for patients, health professionals can effectively learn physical skills [41], technical knowledge and skills [42], as well as communication skills from patients [43]. However, we recognize that these findings often result from sustained or longer educational encounters between patients and health professionals rather than episodic or short 3–5 min digital story compilations.

It is important to note that, in Price et al. [9] and Cueva and colleagues (2016, 2013), the students created their own digital stories that encompassed their experiences as both health professionals and patients or family members of patients. The use of these types of stories is interesting and warrants further investigation. Specifically, it would be interesting to continue exploring if such stories enhance students’ self-awareness, empathy, and compassion as health professionals.

We also recognize that three (18.8%) of the reviewed studies commented on the benefits of training individuals in the creation of digital stories [23, 29, 31]. Researchers long have established that training, regardless of the context or topic, can help maximize participants’ learning and behaviour changes [18]. Thus, it is not surprising that the participants’ in these studies experienced learning and had positive experiences with digital storytelling. While we recognize that offering formal training may not be feasible within resource-limited educational settings, there are a number of free or low-cost e-learning workshops offered by experts in the field that can increase accessibility to training opportunities. In addition, it is commendable that three (18.8%) of the authors discussed the educational benefits of using digital storytelling in combination with other teaching and learning strategies [22, 28, 35]. From an educational perspective, the use of multiple strategies, including the use of reflection, is important for moving from superficial to deep learning as well as preparing students for the complexities of professional life [44].

Finally, it is important to remember that all the reviewed studies relied on selected participants’ self-reported learning and that only two (12.5%) of the studies reported on participants’ self-reported behaviour changes. Although researchers commonly use self-reported measures in HPE research [45], it is possible that the participants in these studies distorted their responses or that the wording of the questions as well as other aspects of the research environments undermined the validity and reliability of the results and interpretations [46]. Thus, these self-reports combined with the mediocre quality of the reviewed studies presents a strong rationale for conducting additional research on digital storytelling in HPE before rendering conclusions about its use and impact on health professionals.

Future studies investigating health professionals’ learning from digital storytelling could include, for example, well-developed knowledge tests, teach backs (e.g., where learners teach portions of material covered in digital stories to their peers to confirm understanding), or high-quality qualitative studies [18]. Similarly, explorations of health professionals’ behaviour changes from digital storytelling could again involve high-quality qualitative studies that include observations of the participants’ post-digital storytelling experiences [18]. Regardless of the future research approaches used, it is essential to include a priori identified bases of comparison (i.e., something the collected data will be compared to) or counterfactuals (i.e., an understanding of what outcomes would have occurred in the absence of the digital stories) in order to appropriately determine the impact of digital storytelling on health professionals’ learning and behaviours [47].

### Limitations

First, while we attempted to identify all pertinent search terms, given the complexity of searching within this topic area, we may have unknowingly omitted keywords or synonyms used for digital storytelling and thus, missed some empirical studies. Second, we ran our last search in December 2016 and as such, we may have missed recently published studies on this topic. Third, the heterogeneity of the reviewed studies limited the type of results presented. Specifically, meta-analysis of outcome data was not feasible. Lastly, the included studies needed to report on at least one outcome from Levels 2 or 3 of The New World Kirkpatrick Model [18] and therefore, empirical studies that reported on other outcomes in regards to digital storytelling and HPE are not represented within our dataset.

## Conclusions

This systematic review included 16 empirical studies. Of these studies, 14 (87.5%) focused solely on health professionals’ learning from digital storytelling and 2 (12.5%) investigated health professionals’ learning as well as their behaviour changes from digital storytelling. Eight (50%) of the digital storytelling studies in HPE occurred within the undergraduate nursing context. The reviewed studies reported on the use of digital storytelling for a variety of different purposes and covered a wide-range of topics. The authors focused on one of the following: (a) health professionals’ learning from creating their own digital stories, (b) health professionals’ learning from the perspectives of both the creators and viewers of the digital stories, (c) health professionals’ learning from listening to authentic patients’ digital stories, (d) health professionals’ learning from ‘patients’ stories created by a third party, and (e) health professionals’ learning from viewing the digital stories of both health professionals and patients/family members. Overall, the co-creation of patients’ digital stories with health professionals as well as the creation and use of health professionals’ own digital stories positively enhanced learning. Patients’ digital stories alone had minimal impact on health professionals’ understanding or knowledge gains. However, all the reviewed studies relied solely on health professionals’ self-reported learning. This review also illustrated the educational value of training individuals in the creation of digital stories as well as the benefits of including digital stories in conjunction with other learning activities. Unfortunately, few studies focused on changes in health professionals’ behaviours as a result of digital storytelling, and those that did focused solely on self-reported behaviour changes. In conclusion, this review highlights the need for additional high-quality research on the use and impact of digital storytelling in HPE.

## Additional files

Additional file 1: MEDLINE search strategy. This file includes the search strategy used to search the database MEDLINE, including the search terms and operators used. (DOCX 12 kb)

Additional file 2: Summary of the Evidence on the Purpose and Impact of Digital Storytelling in Health Professions Education. This file includes a summary of the evidence from the studies included in this systematic review, including the study author, year, context, purpose of using digital storytelling, details, and findings. (DOCX 39 kb)

## Abbreviation

HPE: Health professions education
