# Supplementary material for: Digital storytelling in health professions education: a systematic review
Source: BMC Med Educ. 2018 Sep 10;18:208. doi: 10.1186/s12909-018-1320-1 (PMC6131857; doi:10.1186/s12909-018-1320-1)
Supplement: Supplementary file 1 — MEDLINE search strategy. This file includes the search strategy used to search the database MEDLINE, including the search terms and operators used. (DOCX 12 kb) [file 12909_2018_1320_MOESM1_ESM.docx]

Additional file 1

*MEDLINE Search Strategy*

(((story telling or story* or stories or narrati* or documentar* or memoir* or essay*) adj3 (internet or informatics or digital or multimedia or media or social media or blog* or computer or interactive or video*)) not (((news or media) adj3 (stor* or coverag* or narrativ*)) not (personal adj3 (stor* or narrativ*)))).mp.
