# Supplementary material for: Digital storytelling in health professions education: a systematic review
Source: BMC Med Educ. 2018 Sep 10;18:208. doi: 10.1186/s12909-018-1320-1 (PMC6131857; doi:10.1186/s12909-018-1320-1)
Supplement: Supplementary file 2 — Summary of the Evidence on the Purpose and Impact of Digital Storytelling in Health Professions Education. This file includes a summary of the evidence from the studies included in this systematic review, including the study author, year, context, purpose of using digital storytelling, details, and findings. (DOCX 39 kb) [file 12909_2018_1320_MOESM2_ESM.docx]

Additional file 2

*Summary of the Evidence on the Purpose and Impact of Digital Storytelling in Health Professions Education*

| **Author, year** | **Context** | **Purpose of use** | **Study details** | **Findings** |
| --- | --- | --- | --- | --- |
| Bruno [27] | Undergraduate | To teach medical students about physicians’ responsibilities toward the underserved | - Understand the impact of digital stories on medical students’ attitudes towards healthcare services for the un/underinsured - Understand medical students’ desires to provide care to underserved patients - Randomized-controlled trial - Medical students - N = 895 (494 Intervention group; 401 control group) - Health insurance | - No significant change in attitudes, potentially due to ceiling effect pre-digital stories - Significantly higher proportion of students expressed they wanted to be involved in providing care to those without access |
| Christiansen [28] | Undergraduate | To enhance nursing students’ overall learning | - Understand variations in students’ conceptions of patients’ digital stories - Qualitative - Nursing students - N = 20 - Patients with various health conditions | - Perceived as more powerful and a better learning resource than written stories and situated interactions with patients in the clinical setting; perceived not as powerful as patients directly in the classroom - At times, multi-media form of stories distracted students from the patients’ key messages - Triggered emotional responses among students, making them sad and angry when patient experiences were negative |
| Cueva [32] | Continuing professional development (CPD) | To facilitate Alaskan community health workers’ provision of cancer support in their communities | - Understand community health workers’ perceptions of digital stories as a culturally appropriate health communication intervention and its impact on their learning - Non-experimental - Community health workers - N = 67 completed post-education questionnaire; 24 completed follow-up questionnaire) - Cancer | - Perceived improvement in cancer knowledge - Increased confidence in cancer knowledge and abilities to share cancer information with communities - Reported intentions to embrace healthy behaviors for themselves and others (e.g., encouraging screening, healthy eating, physical activity) - 3-years post, about a third shared their digital stories on cancer with their communities, some attended screening exams, 1 quit smoking and 1 reduced use, some increased their physical activity and changed their eating habits |
| Cueva [33] | CPD | To teach Alaskan community health aids/practitioners and people in their communities about cancer | - Understand the impact of creating digital stories on community health aids/practitioners’ cancer knowledge, attitudes, and health behaviors - Explore how community health aids/practitioners used their stories as health communication interventions in their communities - Mixed methods - Community health aids/practitioners - N = 30 completed post-education questionnaire; 19 completed follow-up questionnaire - N not provided for participation in post-education group sharing or completion of logs - Cancer | - Perceived increase in knowledge acquisition and understanding of cancer - Perceived change in attitudes towards cancer and personal experiences of cancer; more comfortable with speaking about cancer and accepting cancer - Indicated committing to quitting smoking, eating healthier, increasing physical activity, and having screening exams - 6-months post, indicated they had quit smoking, reduced smoking, increased physical activity, were eating healthier, and having screening exams - Participants shared their stories about using digital stories as a health communication intervention with family, friends, patients and coworkers via Facebook, YouTube, other websites, community gatherings, tribal council meetings, health fairs, school classes, and sport tournaments |
| D'Alessandro [21] | Undergraduate | To teach medical students pediatric clinical skills | - Describes the development of a digital storytelling system (computer-based patient simulation with real patient stories) and understand its perceived usability, clarity, quality, and value for medical students - Non-experimental - Medical students - N = 79 - Various health conditions | - Reported improvement in confidence; believed they could begin to evaluate a similar patient and case presentation - Perceived digital stories as memorable |
| Eggenberger [24] | CPD | To teach family-centered care | - Explored how digital stories impact nurses’ perceptions of and confidence in providing family-centered care - Mixed methods - Nurses and family members - N Pre-education = 30 nurses completed pre-education questionnaire; 5 nurses participated in focus group; 35 family members completed questionnaire - N Post-education = 14 nurses completed post-education questionnaire - Critical conditions | - Perceived increase in family-centered knowledge from pre-intervention to post-intervention - Perceived improvement in working with family units - Perceived increase in confidence in providing family-centered care - Participants were satisfied and perceived digital stories as valuable and effective |
| Fenton [34] | Undergraduate | To enhance nursing students’ understanding of pediatric life threatening/chronic health conditions | - Understand nursing students’ perceptions of a digital story as a teaching and learning tool - Non-experimental - Nursing students - N = 40 - Pediatric cancer | - Reported learning about the perspective of a young person with cancer - Reported learning about communication skills - Reported learning about the effects that a cancer diagnosis and its treatment can have on a patient |
| Gazarian [25] | Undergraduate | To teach nursing students about advocacy | - Understand how nursing students’ digital stories on an ethical concern in practice impacted their advocacy role - Quasi-experimental - Nursing students - N = 36 - Health advocacy | - Significant changes in perceptions about their advocacy role as measured by the Protective Nursing Advocacy Scale |
| Hewson [31] | Undergraduate | To facilitate intergenerational learning among older adults and social work students | - Understand the impact of having social work students assist older adults in the creation of digital stories - Understand the use of older adults’ digital stories for intergenerational learning among social work students - Non-experimental - Social work students & older adults - N Social work students = 7 - N Older adults = 7 - Aging | - Social work students and older adults increased their knowledge of digital storytelling and its application to social work - Students reported increased knowledge about working with older adults - Students perceived improvement in their interpersonal skills (e.g., supporting, listening, and helping older adults) - Students reported changes in their understandings of how important it is to share and listen to stories - Older adults reported positive digital storytelling creation experiences |
| Levett-Jones [26] | Undergraduate | To facilitate transformative learning and enhance nursing students’ understanding of concepts such as social justice, person-centered care, and patient safety | - Understand the impact of digital stories on nursing students’ learning of social justice, person-centered care, and patient safety - Non-experimental - Nursing students - N not provided - Various health conditions | - Reported changes in how they perceived the concepts of community and social justice - Reported increased understandings of Aboriginal cultures and refugee issues |
| Loe [30] | Undergraduate | To promote intergenerational community-based learning among undergraduate students in health-related programs and them about aging | - Described the digital storytelling intervention and explored its impact on the students and older adults - Qualitative - Undergraduate students in health-related programs & older adults - N not provided - Aging | - Reported changes in students’ perspectives of older adults - Reported changes in older adults’ perspectives of young adults - Reported changes in students’ attitudes towards aging - Students and older adults developed a relationship through their participation - Reported greater student awareness of the human aging process - Reported greater student awareness of life changes and embracing/preparing for these changes |
| Price [9] | Undergraduate | To enhance nursing students’ learning experience on palliative and end-of-life care | - Nursing students developed their own digital stories about palliative and/or end-of-life care from a personal or professional perspective - Study the impact of digital stories on student nurses’ learning of palliative and end-of-life care and how educators can use digital stories to facilitate peer learning and sharing - Mixed methods - Nursing students - N = 68 - Palliative & end-of-life care | - Perceived increase in understanding of palliative care and end-of-life concepts - Perceived increase in understanding about culture and spirituality and about the uniqueness of each person's and family's end-of-life experience - Helped promote new insights and seeing the value in making interpersonal connections with patients/families |
| Snelgrove [35] | Undergraduate | To help nursing students learn about psychological concepts related to chronic illness | - Understand the impact of patients’ digital stories on nursing students’ knowledge and application of psychological concepts and their understanding of chronic illness and its effects on the patients - Mixed methods - Nursing students & clinician educators - N Nursing students = 29 completed demographic questionnaire; 19 completed attitudinal questionnaire; 0 completed knowledge tests; 4 participated in focus group - N Clinician educators = 5 participated in focus group - Chronic conditions | - 1/4 of the students believed that the digital stories made them more confident in their ability to care for patients with chronic illness - Some students did not recognize link between the digital stories and psychology - Some students perceived an increase in their understanding of the nature of chronic illness, the psychological impact of being diagnosed, and the role of nurses in chronic illness and in enhancing the patient experience - Students valued the authenticity of a real patient telling their story - Educators perceived the digital stories as clear and well-structured - Educators raised ethical challenges of using videos (i.e., access and how to protect) - Educators believed the digital stories raised psychological concepts new to students (e.g., grief and bereavement). |
| Stacey [29] | Undergraduate | To facilitate the transition from student nurse to practicing nurse | - Describes the development of digital stories by newly qualified and practicing nurses to help nursing students transition to professional practice - Describes nursing students’ experiences of listening to the qualified and practicing nurses’ digital stories - Qualitative - Nurses & nursing students - N Nurses = 8 - N Nursing students = 58 - Student-professional transition | - Qualified nurses learned about digital storytelling and their emotions surrounding professional transitions - Encouraged students to self-reflect - Students felt reassured and supported that others had experienced similar transition concerns - Students could relate to the nurses telling their stories |
| Taylor [22] | Undergraduate | To influence midwifery students’ breastfeeding practices | - Understand the impact of digital stories of women’s experiences of breastfeeding on midwifery students - Qualitative - Midwifery students - N = 11 - Breastfeeding | - Reported no new breastfeeding knowledge; instead they said it facilitated their learning on how to support breastfeeding women - Developed a strong desire to improve support for breastfeeding women - Generated emotions, including admiration, sadness and despair for wanting to both improve and defend midwifery practice - Suggested peer support and guided/reflective study as essential components of learning with digital stories |
| Walsh [23] | Graduate | To teach graduate social work students about issues related to diversity and oppression in society | - Describes the course design and the impact of digital storytelling on students’ knowledge of diversity and oppression in society - Qualitative - Social workers - N = 15 - Social oppression/diversity | - Perceived digital storytelling as a useful tool to use in practice to potentially bridge any power differentials between them and clients - Believed the digital stories were important for enriching and diversifying their learning - Digital stories allowed students to self-reflect on the given topics |
